# The Enhanced Liver Fibrosis test maintains its diagnostic and prognostic performance in alcohol-related liver disease: a cohort study

**DOI:** 10.1186/s12876-021-01795-5

**Published:** 2021-06-28

**Authors:** Declan Connoley, Preya Janubhai Patel, Brian Hogan, Sudeep Tanwar, Freya Rhodes, Julie Parkes, Alastair Burt, Jennifer Watkins, William Sievert, William Rosenberg

**Affiliations:** 1 Monash University, Melbourne, Australia; 2 Monash Health, Melbourne, Australia; 3 Institute for Liver and Digestive Health, Division of Medicine, University College London, Royal Free Campus, Rowland Hill Street, Hampstead, London, NW3 2PF UK; 4 The Royal Free London NHS Foundation Trust, London, UK; 5 Primary Care, Public Health and Medical Statistics, University of Southampton, Southampton, UK; 6 Faculty of Health and Medical Sciences, University of Adelaide, Adelaide, Australia

**Keywords:** Serum biomarker panel, Liver fibrosis, Alcohol-related liver disease, Non-invasive testing, Cirrhosis, Diagnosis, Prognosis

## Abstract

**Background:**

Alcohol is the main cause of chronic liver disease. The Enhanced Liver Fibrosis (ELF) test is a serological biomarker for fibrosis staging in chronic liver disease, however its utility in alcohol-related liver disease warrants further validation. We assessed the diagnostic and prognostic performance of ELF in alcohol-related liver disease.

**Methods:**

Observational cohort study assessing paired ELF and histology from 786 tertiary care patients with chronic liver disease due to alcohol (n = 81) and non-alcohol aetiologies (n = 705). Prognostic data were available for 64 alcohol patients for a median of 6.4 years. Multiple ELF cut-offs were assessed to determine diagnostic utility in moderate fibrosis and cirrhosis. Survival data were assessed to determine the ability of ELF to predict liver related events and all-cause mortality.

**Results:**

ELF identified cirrhosis and moderate fibrosis in alcohol-related liver disease independently of aminotransferase levels with areas under receiver operating characteristic curves of 0.895 (95% CI 0.823–0.968) and 0.923 (95% CI 0.866–0.981) respectively, which were non-inferior to non-alcohol aetiologies. The overall performance of ELF was assessed using the Obuchowski method: in alcohol = 0.934 (95% CI 0.908–0.960); non-alcohol = 0.907 (95% CI 0.895–0.919). Using ELF < 9.8 to exclude and ≧ 10.5 to diagnose cirrhosis, 87.7% of alcohol cases could have avoided biopsy, with sensitivity of 91% and specificity of 85%. A one-unit increase in ELF was associated with a 2.6 (95% CI 1.55–4.31, p < 0.001) fold greater odds of cirrhosis at baseline and 2.0-fold greater risk of a liver related event within 6 years (95% CI 1.39–2.99, p < 0.001).

**Conclusions:**

ELF accurately stages liver fibrosis independently of transaminase elevations as a marker of inflammation and has superior prognostic performance to biopsy in alcohol-related liver disease.

**Supplementary Information:**

The online version contains supplementary material available at 10.1186/s12876-021-01795-5.

## Background

Excessive alcohol consumption is the commonest cause of chronic liver disease (CLD) accounting for 5.1% of the global burden of disease [1]. Chronic alcohol use causes hepatic steatosis that progresses to fibrosis in 10–35% and cirrhosis in 10–20% of cases. Increasing alcohol consumption in the USA is leading to rising alcohol related liver disease (ARLD) mortality with alcohol as the cause of half all cirrhosis-related deaths [1–4].

Fibrosis severity has prognostic significance and influences clinical decisions in the management of ARLD [5]. Abstinence is beneficial in all stages of ARLD and may lead to reversal of early fibrotic changes [6] and even in advanced cirrhosis surveillance for the detection and treatment of complications is recommended. Liver biopsy (LB) is the reference standard for fibrosis assessment, but diagnostic accuracy is influenced by sampling error and observer interpretation and it is associated with rare but significant complications [7–10]. Biopsy is not amenable to use in community settings and biopsies cannot be repeated frequently to monitor disease. Thus, there is an exigent need for less invasive tests capable of detecting both cirrhosis and early stages of fibrosis in ARLD.

The Enhanced Liver Fibrosis (ELF) test is a non-invasive test that combines measurements of three markers of hepatic extracellular matrix—procollagen type III N-terminal peptide, tissue inhibitor of metalloproteinase-1 and hyaluronic acid—to generate a unitless numerical score [11]. ELF can be applied to the stratification of patients with liver fibrosis using two thresholds: an upper specific threshold to detect advanced fibrosis or cirrhosis and a lower sensitive threshold to exclude fibrosis [5, 12–15].

ELF performs well as a non-invasive marker of fibrosis in viral hepatitis, NAFLD and cholangiopathies and is a better prognostic marker than biopsy in these aetiologies [5, 16], however there is a paucity of literature regarding the use of ELF in ARLD [11, 17]. Although concern exists that ongoing alcohol consumption may affect the levels of individual ELF constituents [18], a recent study validated the use of ELF in detecting significant and advanced fibrosis and cirrhosis in ARLD [13]. Further investigation in an ARLD cohort is necessary to validate these findings.

We investigated the diagnostic performance of ELF in ARLD compared to its performance in aetiologies in which ELF has been previously validated to assess non-inferiority, and to determine the effect, if any, of inflammation on ELF score. We also examined the prognostic performance of ELF in ARLD. We hypothesised that the performance of ELF in ARLD would be non-inferior to aetiologies other than alcohol.

## Methods

**Design:** Cohort Study.

**Setting:** Tertiary care.

### Participants

Data were pooled from three observational studies which prospectively recruited patients undergoing planned LB for the investigation of CLD in UK secondary care centres.

Cohort-1 (n = 5) included patients recruited to a single-centre observational cohort study investigating the diagnostic performance of ELF in ARLD. Cohort-2 was derived from 921 patients with mixed-aetiology CLD recruited from 13 centres between 1998 and 2000 [11]. Cohort-3 included 97 patients undergoing a transjugular LB for investigation of CLD [19, 20] from 2011 to 2013 see (Table 1).

**Table 1:** Participants’ clinical characteristics—Cohort-NA versus Cohort-A

	Cohort-NA (n = 705)	Cohort-A (n = 81)	P value
Ishak stage [n (%)]			
0	143 (20.1)	5 (6.2)	< 0.001
1	164 (23.1)	7 (8.6)	
2	110 (15.5)	6 (7.4)	
3	99 (13.9)	4 (4.9)	
4	64 (9.0)	5 (6.2)	
5	51 (7.2)	10 (12.3)	
6	75 (10.6)	44 (54.3)	
ELF score [n(%)]			
≧ 8.3	379 (53.8)	74 (91.4)	< 0.001
≧ 9.8	128 (18.2)	59 (72.8)	
≧ 10.5	79 (11.2)	49 (60.5)	
≧ 11.3	40 (5.7)	38 (46.9)	
Male gender [n (%)]	456 (64.7)	55 (67.9)	0.595
Age [median (IQR)]	43.0 (35.0–54.5)	50.0 (41.5–57.5)	< 0.001
ALT (IU/L) [median (IQR)]*	55 (31–93)	36 (23–66)	< 0.001
Platelet count (10^9^/L) [median (IQR)]^	203 (159–249)	162 (105–220)	< 0.001
Bilirubin (umol/L) [median (IQR)]^+^	12 (9–16)	20 (11–102)	< 0.001

Cohort-A n = 79; Cohort-NA n = 682. ^Cohort-A n = 81; Cohort-NA n = 697. ^+^Cohort-A n = 74; Cohort-NA n = 650

### Inclusion/exclusion criteria

Patients recruited to all 3 studies were aged between 18 and 75 years undergoing a planned liver biopsy.

Exclusion criteria included any extrahepatic fibrotic disorder including rheumatoid arthritis, systemic sclerosis and pulmonary fibrosis; no available histological staging (n = 6) or ELF score (n = 2) or if fibrosis was due to an extra-hepatic aetiology (n = 229). Patients were excluded from study-2 if they were taking aspirin, had cardiovascular disease, cancer, decompensated cirrhosis (Child–Pugh C), HCC or drug induced liver injury.

### Prognostic data

Prognostic data for a subset of 64 ARLD subjects (Study-2 = 49, Study-3 = 15) were available for a median period of 6.4 years (range 0–9.1; IQR 2.8–8.5) and were interrogated for Liver Related Events (LRE), defined as complications of portal hypertension, liver cancer, liver transplantation or death. Further details regarding data collection have been published previously [5, 20].

### ELF test

ELF scores were calculated from sera collected ≤ 14 days prior or at the time of biopsy. ELF markers were measured individually using the Siemens Immuno-1 or Advia Centaur XP platform according to manufacturer’s instructions (Siemens Healthineers). Technicians performing the ELF test were blinded to histological assessment. Manufacturer and literature defined cut-offs were used to determine moderate fibrosis (< 8.3 and < 9.8) and cirrhosis (≧ 9.8, ≧ 10.5 and ≧ 11.3) [5, 11, 13–15].

### Histology

Biopsies were processed using standard techniques and read by two expert hepatic pathologists (AB or JW) blinded to ELF scores. Biopsies were required to be ≧ 15 mm with ≧ 9 portal tracts. Fibrosis was staged using the Ishak scale from F0-F6, with modifications made to reflect the distribution of fibrosis in aetiologies other than chronic viral or autoimmune hepatitis. Specifically in ARLD, perivenular and pericellular fibrosis replaced portal and periportal fibrosis. Ishak stages ≧ 3 were classified as moderate fibrosis and stages ≧ 5 as cirrhosis for binary outcome assessment.

### Statistical analyses

Data analyses were conducted using SPSS Inc version 23.0 (College Station TX: StatCorp LP; 2013) with the exception of De Long’s test and the Obuchowski Method (R version 3.3.3). All p values were 2-sided and statistical significance was set at alpha = 0.05.

#### Diagnostic performance

Area under receiver operator curve (AUROC) was used to compare diagnostic accuracy between ELF and LB. De Long’s test was used to assess significance of differences in AUROCs [21]. The Obuchowski measure was used to calculate a weighted AUROC (ordROC) to more appropriately compare ELF to the ordinal variable of Ishak staging and account for the spectrum effect. The Obuchowski measure is explained in more detail (see Additional file 1) [22, 23]. Sensitivity, specificity, positive predictive value (PPV), and negative predictive value (NPV) for relevant cut-offs were calculated [24]. APRI and AST:ALT ratio were calculated in a subset of patients. Two ELF thresholds (< 8.3 and ≧ 9.8 in F ≧ 3 and < 9.8 and ≧ 10.5 in F ≧ 5) were used to determine the number of biopsies that might be avoided in patients with ARLD (Cohort-A), assuming biopsy would be limited to resolve cases in which the scores fell between thresholds.

#### Prognostic performance

Prognostic data were assessed for LREs (previously defined) using Cox Proportional-Hazard models adjusted for age and sex, Kaplan–Meier survival curves for LRE-free survival and AUROC curves for ELF and biopsy based on event occurrence at specific follow-up intervals.

#### Effect of aminotransferase elevation as a marker of inflammation

A univariate binomial logistic regression analysis was performed in a derivation cohort of patients with CLD (excluding ARLD) to identify potential predictors of cirrhosis and ELF ≧ 10.5 and ≧ 11.3. The multivariate analyses included age, sex, ALT, platelet count, bilirubin and ELF or Ishak stage (as appropriate). Variables with p values less than 0.250 were included in a backwards multiple logistic regression with stepwise selection to identify factors influencing the outcome. Factors which remained significant were run in a validation cohort of ARLD to determine if similar factors influenced the outcome.

## Results

### Baseline characteristics

**Fig. 1:**
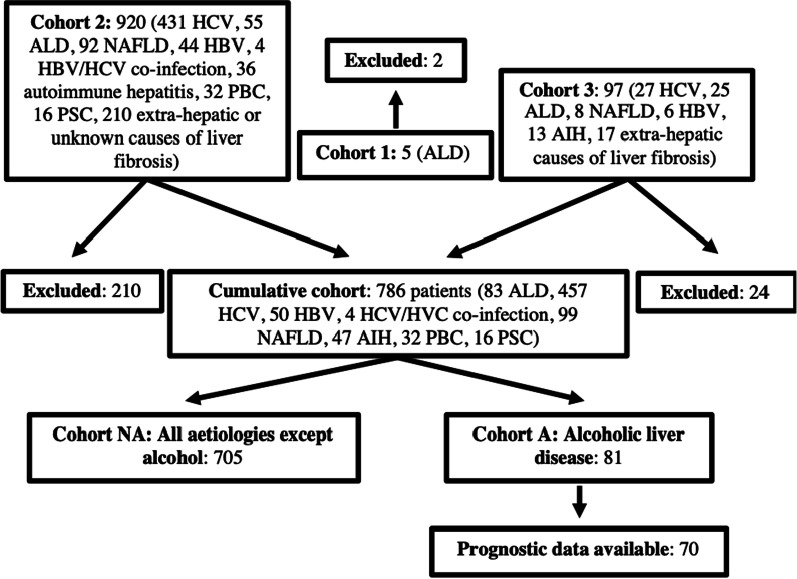
Flow diagram depicting the grouping of cohorts. The ‘cumulative cohort’ included 10.7% alcoholic liver disease

Paired histology and ELF scores were available for 786 patients who met inclusion criteria, 81 with ARLD (Cohort-A) and 705 with CLD due to other aetiologies (Cohort-NA). Cohort-NA was made up of 457 hepatitis C, 50 hepatitis B, 4 hepatitis C/hepatitis B co-infection, 99 NAFLD, 47 autoimmune hepatitis, 32 primary biliary cholangitis and 16 primary sclerosing cholangitis (Fig. 1). Differences in demographic and clinical statistics between cohorts are summarised in Table 1.

The spectrum of disease was significantly more advanced in Cohort-A than Cohort-NA. In Cohort-A 54 (66.7%) patients were F ≧ 5 and 63 (77.8%) were F ≧ 3 compared to 126 (17.9%) were cirrhotic (F ≧ 5) and 289 (41.0%) had moderate fibrosis (F ≧ 3) in Cohort-NA (p < 0.001). A greater proportion of patients had probable or definite cirrhosis (F6) in Cohort-A (81.5%) compared to Cohort-NA (59.5%).

### Diagnostic utility of ELF

**Fig. 2:**
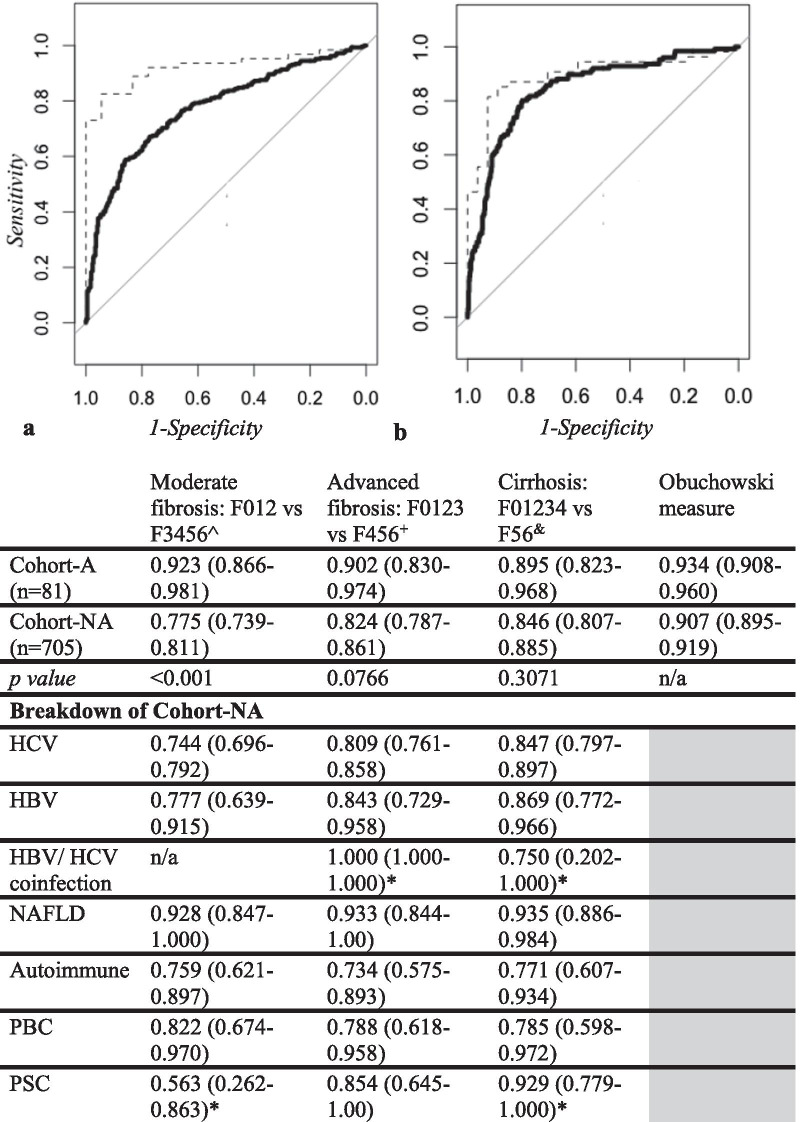
AUROC Curves for Cohort-A (dashed line) and Cohort-NA (solid line) for (**a**) cirrhosis (F ≧ 5) and (**b**) moderate fibrosis (F ≧ 3). P values indicate significance of difference between aetiologies (De Long’s test). All values are expressed as % (95% CI). *p > 0.05 ^F012[n(Cohort-A) = 18; n(Cohort-NA) = 416] versus F3456[n(Cohort-A) = 63; n(Cohort-NA) = 289]; + F0123[n(Cohort-A) = 22; n(Cohort-NA) = 515] versus F456[n(Cohort-A) = 59); n(Cohort-NA) = 190]; &F01234[n(Cohort-A) = 27; n(Cohort-NA) = 579] versus F56[n(Cohort-A) = 54; n(Cohort-NA) = 126). Penalty functions (Obuchowski) were assigned proportional to the difference in Ishak units between stages as follows: 0.17, 0.33, 0.50, 0.67, 0.83 and 1.00 for differences of 1, 2, 3, 4, 5, and 6 stages

ELF correlated well with histology, with Spearman’s coefficient values of 0.695 for Cohort-A and 0.535 for Cohort-NA (p < 0.01). Overall diagnostic performance of ELF was excellent (ordROC = 0.934 (95% CI 0.908–0.960) in Cohort-A; 0.907 (95% CI 0.895–0.919) in Cohort-NA) (Fig. 2).

#### Diagnostic accuracy of ELF for moderate fibrosis

The diagnostic accuracy of ELF for F ≧ 3 was significantly greater in ARLD than in other pathologies with AUROC for Cohort-A = 0.923 (95% CI 0.866–0.981) compared 0.775 (95% CI 0.739–0.811) for Cohort-NA (p < 0.001) (Fig. 2).

In Cohort-A, a threshold of ≧ 8.3 yielded the sensitivity of 97% for moderate fibrosis (F ≧ 3). ELF ≧ 9.8 yielded a specificity of 83%. The corresponding figures in Cohort-NA were 78% and 38% respectively (Table 2).

**Table 2:** diagnostic test probabilities for moderate fibrosis (F ≧ 3) and cirrhosis (F ≧ 5) using multiple thresholds for ELF, APRI and AST:ALT ratio

Aetiology	Stage		Threshold	Sensitivity	Specificity	PPV	NPV	LR +	LR −
Cohort-A	F ≧ 3	ELF	8.3	97 (89–100)	28 (10–53)	82 (78–86)	71 (35–92)	1.34 (1.00–1.79)	0.11 (0.02–0.54)
			9.8	88 (77–95)	83 (59–96)	95 (86–98)	68 (51–82)	5.33 (1.89–15.04)	0.13 (0.06–0.28)
		APRI	< / = 0.5	80 (65–91)	45 (17–77)	85 (76–91)	38 (20–61)	1.48 (0.84–2.58)	0.43 (0.17–1.05)
			> 1.5	39 (24–56)	73 (39–94)	84 (65–94)	24 (17–33)	1.43 (0.51–4.04)	0.84 (0.54–1.30)
	F ≧ 5	ELF	9.8	91 (80–97)	63 (42–81)	83 (75–89)	77 (58–89)	2.45 (1.49–4.04)	0.15 (0.06–0.36)
			10.5	85 (73–93)	89 (71–98)	94 (84–98)	75 (61–85)	7.67 (2.62–22.41)	0.17 (0.09–0.32)
			11.3	67 (53–79)	93 (76–99)	95 (82–99)	58 (48–67)	9.00 (2.34–34.61)	0.36 (0.24–0.53)
		APRI	< / = 1	59 (42–75)	80 (52–96)	88 (72–95)	44 (33–56)	2.97 (1.04–8.47)	0.51 (0.32–0.81)
			> 2	35 (20–53)	87 (60–98)	87 (62–96)	35 (28–42)	2.64 (0.67–10.29)	0.75 (0.55–1.02)
		AST:ALT ratio	> 1	86 (71–95)	50 (23–77)	82 (72–88)	58 (35–79)	1.72 (1.00–2.96)	0.28 (0.11–0.73)
Cohort-NA	F ≧ 3	ELF	8.3	78 (72–82)	63 (58–67)	59 (56–62)	80 (76–83)	2.08 (1.81–2.39)	0.36 (0.29–0.45)
			9.8	38 (32–44)	95 (93–97)	85 (78–90)	68 (67–71)	8.26 (5.19–13.13)	0.65 (0.60–0.72)
		APRI	< / = 0.5	62 (55–68)	71 (66–76)	62 (57–66)	71 (67–74)	2.12 (1.74–2.58)	0.54 (0.45–0.64)
			> 1.5	22 (17–28)	94 (91–97)	75 (64–83)	62 (60–63)	3.96 (2.39–6.57)	0.82 (0.76–0.88)
	F ≧ 5	ELF	9.8	60 (50–68)	91 (88–93)	59 (51–66)	91 (89–92)	6.50 (4.85–8.73)	0.45 (0.36–0.55)
			10.5	37 (29–46)	94 (92–96)	59 (49–69)	87 (86–89)	6.75 (4.50–10.13)	0.66 (0.58–0.76)
			11.3	24 (17–32)	98 (97–99)	75 (60–87)	86 (84–86)	13.79 (6.72–26.84)	0.78 (0.71–0.86)
		APRI	< / = 1	49 (39–59)	86 (82–89)	47 (39–54)	87 (85–89)	3.52 (2.61–4.74)	0.59 (0.49–0.71)
			> 2	25 (17–34)	95 (93–97)	56 (43–69)	84 (82–85)	5.22 (3.07–8.88)	0.79 (0.71–0.88)
		AST:ALT ratio	> 1	32 (23–41)	87 (83–90)	37 (29–46)	84 (82–86)	2.43 (1.68–3.50)	0.78 (0.68–0.90)

AUROCs: *Cirrhosis:* APRI Cohort-NA = 0.715 (95% CI 0.655–0.774, p < 0.001); Cohort-A = 0.753 (95% CI 0.612–0.895, p < 0.01)

AST:ALT ratio: Cohort-NA = 0.634 (95% CI 0.571–0.697, p < 0.001); Cohort-A = 0.788 (95% CI 0.657–0.919, p < 0.005)

*Moderate fibrosis:* APRI: Cohort NA = 0.675 (95% CI 0.629–0.721); Cohort A = 0.692 (95% CI 0.514–0.869, p = 0.053)

As a specific marker for Cirrhosis, the AST: ALT ratio is uninformative in ALD

^*^All values are expressed as % (95% CI). All results significant to the p < 0.001 level

A total of 66 Cohort-A (81.4%) subjects had an ELF score < 8.3 or ≧ 9.8 and could have avoided biopsy, with 92.4% of these being correctly classified. Using the same thresholds 454 (64.4%) Cohort-NA subjects could have avoided biopsy, with 81.5% correctly classified.

#### Diagnostic accuracy of ELF score for cirrhosis

No significant differences between cohorts were found in the diagnostic accuracy of ELF for cirrhosis: AUROC Cohort-A = 0.895 (95% CI 0.823–0.968); Cohort-NA = 0.846 (95% CI 0.807–0.885) (p = 0.307) (Fig. 2).

In Cohort-A, ELF ≧ 9.8 detected cirrhosis (F ≧ 5) with a sensitivity of 91% while a threshold of ≧ 10.5 yielded a specificity of 89% (Table 2). In Cohort-NA, the same thresholds performed with a sensitivity of 60% and specificity of 94%.

Combining these two cut-offs for cirrhosis, 71 patients (87.7%) in Cohort-A could have avoided biopsy with an accuracy of 88.7%. In Cohort-NA, 656 (93.0%) could have avoided biopsy with 87.3% correctly classified.

#### Performance of APRI and AST:ALT ratio

APRI (Cohort-A, n = 52; Cohort-NA, n = 557) and AST:ALT ratio (Cohort-A, n = 50; Cohort-NA, n = 545) were calculated in patients where data were available (Table 2). In ARLD, APRI diagnosed F ≧ 3 with 80% sensitivity and 73% specificity and F ≧ 5 with 59% sensitivity and 87% specificity. The AST:ALT ratio, detected cirrhosis with 86% sensitivity and 50% specificity. Both tests performed better in Cohort-NA than Cohort-A (Table 2).

### Factors associated with cirrhosis

Multivariable logistic regression analyses identified ELF [OR = 2.166 (95% CI 1.808–2.595)] (p < 0.001), platelet count [OR = 0.992 (95%CI 0.988–0.996)] (p < 0.01) and ALT > 2xULN [OR = 1.869 (95%CI 1.073–3.254)] (p < 0.05) as independent predictors of cirrhosis in Cohort-NA. Validation of this model in Cohort-A showed that only ELF and platelets were statistically significant markers of cirrhosis (Table 3). Adding ALT and platelets compared to ELF alone did not improve accuracy. In ARLD, a one-unit increase in ELF was independently associated with 2.6 times greater odds of cirrhosis, similar to other aetiologies [5, 12, 25].

**Table 3:** Multivariate stepwise logistic regressions for (A) histologically staged cirrhosis and (B) ELF ≧ 10.5

	Derivation cohort: Cohort-NA		Validation cohort: Cohort-A	
	ORs (95% CI)	p value	ORs (95% CI)	p value
(A) *Cirrhosis*				
ELF	2.17 (1.81–2.60)	< 0.001	2.58 (1.55–4.31)	< 0.001
ALT ≧ 2xULN	1.87 (1.07–3.25)	0.027	0.13 (0.02–1.06)	0.057
Platelets	0.992 (0.988–0.996)	< 0.001	0.984 (0.973–0.996)	< 0.01
(B) *ELF ≧ 10.5*				
Ishak 5/6	5.64 (3.21–9.89)	< 0.001	50.68 (11.63–220.95)	< 0.01
Age	1.05 (1.03–1.08)	< 0.001	1.01 (0.96–1.07)	0.637
Platelets	0.991 (0.986–0.996)	< 0.001	0.999 (0.990–1.008)	0.751

### Prognostic performance in Cohort-A

The incidence of LREs increased during the censor period with increasing ELF score.

**Fig. 3:**
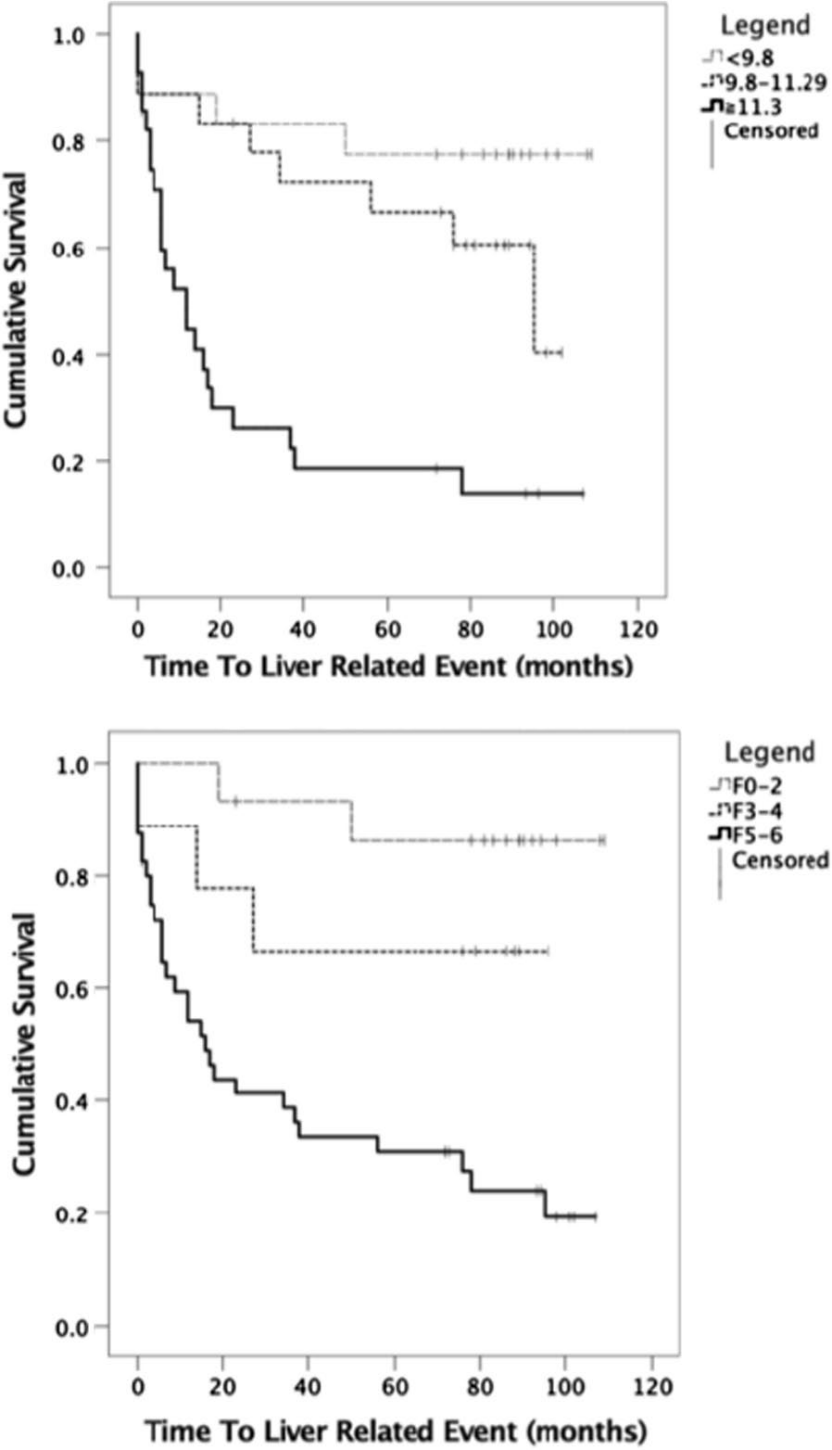
Kaplan–Meier survival curves at 2, 4, 6 and 8 years for ELF thresholds (**a**) and Ishak stage (**b**)

Using Cox Proportional-Hazard modelling adjusted for age and gender, each unit increase in ELF was associated with a 1.44 times increased risk of LRE (95% CI 1.25–1.66, p < 0.001) (Table 4). Fully adjusted HRs for LREs demonstrated a graded response, although differences were only statistically significant when comparing the highest tertile to the lowest tertile: compared to ELF < 9.8, HR was 1.49 (95% CI 0.287–7.74) for ELF 9.8–10.49, 3.84 (95% CI 0.90–16.39) for ELF 10.5–11.29 and 10.24 (95% CI 2.97–35.27) for ELF ≧ 11.3. Crude unadjusted Kaplan–Meier plots reinforced this graded relationship between baseline ELF and LREs (Log rank test (Mantel–Cox) p < 0.01) (Fig. 3).

**Table 4:** Hazard ratios for liver related events in Cohort-A with available prognostic data (n = 64) derived from Cox Proportional-Hazards model analyses

	ELF cut-offs	Unadjusted HRs (95% CI)	P value	HRs adjusted for age and sex (95% CI)	P value
ELF as continuous (overall)	n/a	1.39 (1.22–1.59)	< 0.001	1.44 (1.25–1.66)	< 0.001
ELF split into four categories	< 9.8 (reference)	1	1	1	1
	9.8–10.49	1.68 (0.34–8.31)	0.528	1.49 (0.287–7.74)	0.634
	10.5–11.29	4.45 (1.09–10.05)	< 0.05	3.84 (0.90–16.39)	0.069
	≧ 11.3	10.00 (2.97–33.68)	< 0.001	10.24 (2.97–35.27)	< 0.001
ELF split into two categories	< 10.5	1	1	1	1
	≧ 10.5	6.49 (2.67–15.78)	< 0.001	6.42 (2.63–15.24)	< 0.001
Ishak split into three categories	< 3 (reference)	1	1	1	1
	3–4	2.99 (0.50–17.88)	0.231	3.05 (0.51–18.34)	0.222
	5–6	9.88 (2.35–41.56)	< 0.005	10.45 (2.46–44.34)	< 0.05
Ishak split into two categories	0–4	1	1	1	1
	5–6	5.93 (2.28–15.39)	< 0.001	6.202 (2.37–16.21)	< 0.001

Logistic regression, adjusted for age and gender, showed a one unit increase in ELF was associated with a 2.0 times greater risk of an LRE within 6 years (95% CI 1.39–2.99, p < 0.001). After adjustment for biopsy, ELF remained a significant predictor of LREs at 6 years (OR 1.82, 95% CI 1.169–2.83, p < 0.01), indicating ELF predicts LREs independently of biopsy.

ELF was not significantly better than histology in predicting LREs at 6 years: AUROC = 0.816 (95% CI 0.713–0.920, p < 0.001) for ELF compared to 0.709 (95% CI 0.589–0.829, p < 0.001) for histology (p = 0.057). However ELF predicted all-cause mortality at 6 years better than biopsy (p < 0.05): ELF AUROC = 0.733 (95% CI 0.645–0.861, p < 0.05) and biopsy = 0.600 (95% CI 0.470–0.730, p = 0.194) (Table 5). Hazard ratios and AUCROC for liver related events and all-cause mortality for Cohorts 2 and 3 are presented in Additional file 3.

**Table 5:** AUROC for ELF and biopsy predicting clinical events at different survival times

	AUROC at 6 years (95% CI)	AUROC at 7 years (95% CI)	AUROC at 8 years (95% CI)
Liver related event			
ELF	0.816 (0.713–0.920)	0.844 (0.750–0.938)	0.847 (0.754–0.940)
Biopsy	0.709 (0.589–0.829)	0.740 (0.622–0.858)	0.756 (0.640–0.873)
p value*	0.057	0.065	0.105
All-cause mortality			
ELF	0.733 (0.645–0.861)	0.722 (0.591–0.852)	0.722 (0.591–0.852)
Biopsy	0.600 (0.470–0.730)	0.596 (0.464–0.728)	0.596 (0.464–0.728)
p value*	0.032	0.035	0.035

Liver related events at 6 years, 7 and 8 years were 32, 34 and 35 respectively

All-cause Mortality at 6 years, 7 and 8 years were 23, 26 and 26 respectively

^*^p values determined by De Long’s method

## Discussion

In this study of 81 ARLD patients ELF was non-inferior compared to LB in the identification of advanced fibrosis and cirrhosis, and in determining prognosis in ARLD compared to aetiologies other than alcohol [13, 26, 27]. ELF maintained its diagnostic accuracy across all stages of fibrosis with an ordROC of 0.934.

Previously defined thresholds of < 8.3 and ≧ 9.8 for F ≧ 3 and ≧ 9.8 and ≧ 10.5 for F ≧ 5 performed as well in ARLD as in previous validations in aetiologies other than alcohol [5, 11, 13–15].

Although there were few patients with low ELF scores, a score of < 8.3 had high sensitivity (97%) to rule out moderate fibrosis (F ≧ 3) [15]. Similarly ELF ≧ 10.5 had high specificity for diagnosing cirrhosis was sufficiently specific to diagnosing cirrhosis (89%), and justify commencing surveillance programs for the complications of cirrhosis without confirmatory biopsy or orthogonal tests. Prognostic validations in this cohort further support commencement of screening at these thresholds.

ELF scores have an advantage over histological staging in that they maintain a continuous relationship to risk such that a one-unit increase in ELF score corresponds to a twofold increase in the risk of a LRE at 6 years. Thus the difference between ELF scores of 8.4 and 9.4 is clinically meaningful, despite both scores falling between the 8.3–9.8 thresholds encompassing histological stage F3. In our study, the adjusted OR for LRE at 6 years was 2.0 (95% CI 1.39–2.99, p < 0.001), indicating that prognostic performance of ELF in ARLD appears to be non-inferior to previous studies in other aetiologies in which OR range from 1.5 to 3.5 [25].

Risk stratification revealed the graded prognostic value of ELF (Fig. 3; Table 4). Although statistically significant differences were only seen between high and low risk groups, a small number of events in the moderate risk group (n = 3) may explain this. The relatively low number of LREs in our cohort limits the accuracy of this survival analysis, however it is consistent in suggesting non-inferiority of ELF in ARLD [5, 16, 19, 25].

Compared to the other parameters evaluated, ELF was the only clinically significant predictor of cirrhosis in both Cohort-A and Cohort-NA and was associated with fibrosis severity independently of ALT, bilirubin, age, platelets and gender. The addition of parameters incorporated in simple fibrosis panels did not improve ELF performance. Although this study did not specifically enrol patients with alcoholic hepatitis or active drinkers these findings suggest ELF is not influenced by elevated aminotransferase levels, a surrogate marker of inflammation.

Use of FIB-4 and ELF to risk stratify patients with NAFLD in primary care is both clinically effective and cost-effective [28]. The performance of ELF in ARLD supports its use to identify patients with advanced liver fibrosis in similar pathways for patients with alcohol use disorders in primary care—an additional file suggested a pathway of care to facilitate this (Additional file 2). However the use of simple serum biomarker panels, such as APRI or FIB-4, may be inappropriate, given their poor performance in ARLD in this and previous analyses [26, 27]. Instead, ELF alone could risk-stratify patients and has been shown to be theoretically cost-effective [29].

### Limitations

The lack of documented alcohol histories prevented analysis of the impact of recent drinking on the performance of ELF. Whether patients were abstinent following assessment with ELF and biopsy would impact on prognosis and was also not recorded. However, the prognostic performance of both ELF and biopsy would have been similarly affected by this behaviour and the predictive value of each at the point of assessment remains valid. It would be valuable to explore the change in ELF in response to abstinence and correlation with outcomes, whereby ELF may be of value as a biofeedback tool.

Histological staging is an imperfect reference standard due to sampling and observer errors limiting the measured performance of the comparator test. Also reliance on biopsy as a reference standard introduces spectrum bias as biopsy is normally restricted to patients suspected of having advanced fibrosis. The pooled patient groups constituting Cohort-A are unlikely to reflect the spectrum of disease in primary care and validations in this setting, where a greater proportion of patients will be pre-cirrhotic, are required [11, 13, 14]. However the results after adjusting for spectrum effect using the Obuchowski method and validation of ELF thresholds against prognostic outcomes suggests that spectrum bias did not greatly influence ELF performance.

Whilst Cohort-NA was heterogenous, only aetiologies in which ELF is well validated were included. Further, subgroup analyses of Cohort-NA demonstrated good performance across aetiologies, which implies that non-inferiority in Cohort-A was not due to heterogeneity in the comparator group.

Using aminotransferase levels as a surrogate for liver inflammation we found no evidence that ELF scores were affected by inflammation. This is of practical value as ELF test results are usually interpreted with ALT results available but without liver histology. However further work will be required to establish the performance of ELF in patients with alcoholic hepatitis who tend to have more markedly elevated transaminases.

Use of Ishak staging in ARLD is unconventional, however this system was chosen as the single staging used for the whole study in which the single commonest diagnosis was hepatitis C. Staging system scores were harmonized to capture ARLD pathology as described in the methods and dichotomisation of staging into two groups reduces the errors introduced by using other systems. Use of a single pathologist to read all biopsies would have been preferred but was not possible. ELF constituent analytes are stable under a range of storage conditions and ELF score is not significantly impacted by changes in single analyte concentrations given its logarithmic algorithm [30]. This is reassuring that ELF scores are accurate and reliable despite samples being collected at multiple centres during different time periods.

## Conclusions

We found that ELF performed well as a non-invasive marker to stage moderate and advanced fibrosis and as a prognostic marker of fibrosis severity in ARLD and is not influenced by elevated aminotransferase levels implying that it is not affected by hepatic inflammation. Using two thresholds for ELF (< 8.3 and ≧ 10.5), fibrosis severity can be stratified to reduce the need for biopsy and improve diagnostic certainty and the identification of patients requiring specialist involvement. We found evidence that ELF test is of considerable prognostic value in ARLD. This study provides further grounds for the evaluation of ELF in the management of patients with ARLD in primary and secondary care.

## Supplementary Information

**Additional file 1.** Explanation of the Obuchowski measure: A more in depth explanation of a less common statistical method used to calculate the overall performance of the Enhanced Liver Fibrosis test.

**Additional file 2.** Suggested care pathway for the use of ELF in primary care to stratify those at risk of alcohol related liver disease. A suggested pathway for improved identification and stratification of patients with excess alcohol consumption using multiple cut-offs of ELF.

**Additional file 3.** Unadjusted Hazard Ratios, and Hazard Ratios adjusted for age and sex, for liver related events are presented for Cohorts 2 and 3 for ELF as a continuous variable, for ELF at different cut-offs and for Ishak categories. AUROC for liver related events and all-cause mortality are presented for ELF and histological staging for Cohort 2 and Cohort 3. The numbers of subjects in the cohorts were insufficient to allow meaningful Kaplan-Meier Survival analyses or logistic regression analyses for cohorts 2 or 3.

## Data Availability

The datasets used and/or analysed during the current study are available from the corresponding author on reasonable request.

## Abbreviations

ARLD: Alcohol-related liver disease
AUROC: Area under receiver operating characteristic curve
CLD: Chronic liver disease
ELF: Enhanced Liver Fibrosis test
HA: Hyaluronic acid
HCC: Hepatocellular carcinoma
HR: Hazard ratio
LB: Liver biopsy
LRE: Liver related event
NPV: Negative predictive value
OR: Odds ratio
OrdROC: Obuchowski measure
PIIINP: Amino-terminal propeptide of procollagen type III
PPV: Positive predictive Value
TIMP-1: Tissue inhibitor of metalloproteinase-1
