# Supplementary material for: The Enhanced Liver Fibrosis test maintains its diagnostic and prognostic performance in alcohol-related liver disease: a cohort study
Source: BMC Gastroenterol. 2021 Jun 28;21:268. doi: 10.1186/s12876-021-01795-5 (PMC8240375; doi:10.1186/s12876-021-01795-5)
Supplement: Supplementary file 1 — Additional file 1. Explanation of the Obuchowski measure: A more in depth explanation of a less common statistical method used to calculate the overall performance of the Enhanced Liver Fibrosis test. [file 12876_2021_1795_MOESM1_ESM.docx]

# Additional File 1: Explanation of the Obuchowski measure:

**Title:** The Enhanced Liver Fibrosis test maintains its diagnostic and prognostic performance in alcohol-related liver disease: A cohort study.

**Authors:** Declan Connoley^1,2, 3,4^, Preya Janubhai Patel^3, 4^, Brian Hogan^3^, Sudeep Tanwar^3^, Freya Rhodes^3, 4^, Julie Parkes^5^, Alastair Burt^6^, Jennifer Watkins^3^, William Sievert^1,2^, William Rosenberg^3,4^

Given AUROC requires a binary reference standard, Obuchowski measure was used to calculate a weighted AUROC (ordROC) to more appropriately compare non-invasive markers to the ordinal variable of Ishak staging and account for the spectrum effect(263). The Obuchowski measure is the probability that two randomly chosen patient samples from different stages will be correctly ranked by a test. It is essentially a multinomial version of AUROC, whereby pair-wise comparisons are completed between stages and weighted to take into account the distance between stages, with a penalty for misclassifying. We defined a penalty function proportional to the difference in Ishak units between stages as follows: 0.17 when difference between stages was 1, 0.33 when difference was 2, 0.50 when difference was three, 0.67 when difference was 4, 0.83 when difference was 5 and 1.00 when difference was 6. Obuchowski interpretation is analogous to AUROC in that approximation to 1 defines an increasingly better test.
