# Supplementary material for: The Enhanced Liver Fibrosis test maintains its diagnostic and prognostic performance in alcohol-related liver disease: a cohort study
Source: BMC Gastroenterol. 2021 Jun 28;21:268. doi: 10.1186/s12876-021-01795-5 (PMC8240375; doi:10.1186/s12876-021-01795-5)
Supplement: Supplementary file 2 — Additional file 2. Suggested care pathway for the use of ELF in primary care to stratify those at risk of alcohol related liver disease. A suggested pathway for improved identification and stratification of patients with excess alcohol consumption using multiple cut-offs of ELF. [file 12876_2021_1795_MOESM2_ESM.docx]

# Additional File 2: A suggested pathway to identify and risk stratify patients at risk of ARLD in primary care.

**Title:** The Enhanced Liver Fibrosis test maintains its diagnostic and prognostic performance in alcohol-related liver disease: A cohort study.

**Authors:** Declan Connoley^1,2, 3,4^, Preya Janubhai Patel^3, 4^, Brian Hogan^3^, Sudeep Tanwar^3^, Freya Rhodes^3, 4^, Julie Parkes^5^, Alastair Burt^6^, Jennifer Watkins^3^, William Sievert^1,2^, William Rosenberg^3,4^


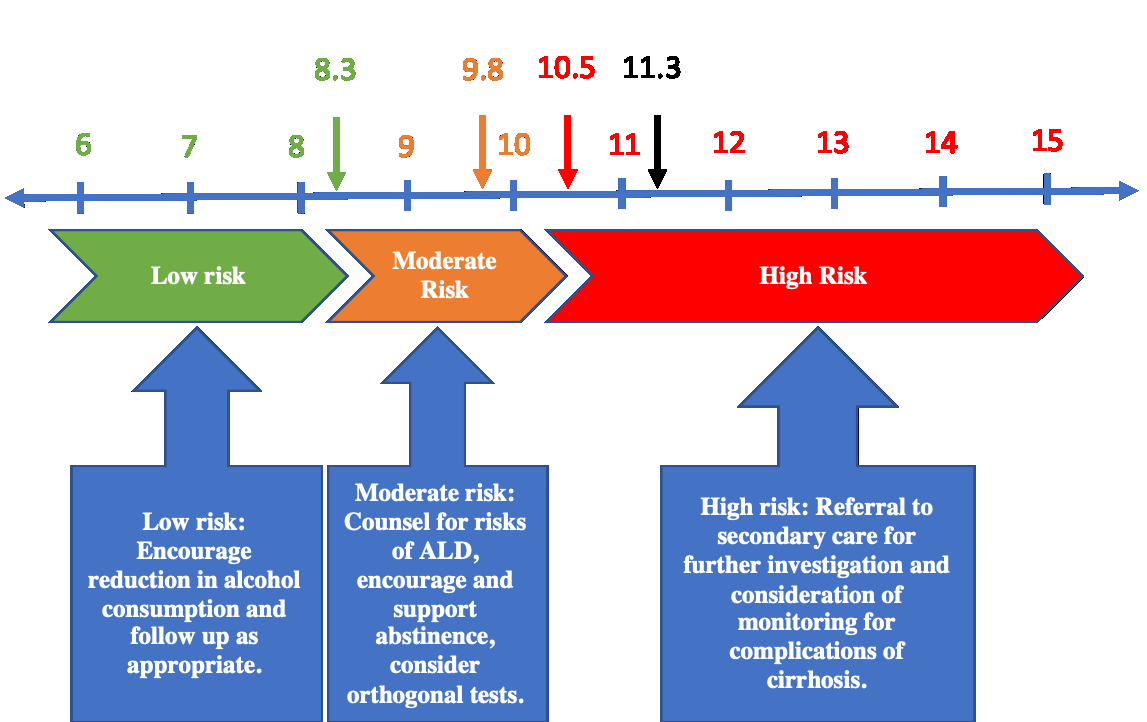


We suggest a pathway for improved identification and stratification of patients with excess alcohol consumption using cut-offs of <8.3 to exclude moderate fibrosis (sensitivity 97%, 0% LREs after 6 years) and ≧10.5 to rule in cirrhosis (specificity 89%, 63% LREs after 6 years). Accordingly, stratification of patients into low (<8.3), moderate (8.3-10.49) or high (≧10.5) risk groups could improve recognition of otherwise silent ARLD and advise health practitioners on ongoing management.

Those with ‘low risk’ ELF are unlikely to have cirrhosis and could be managed in general practice. Given ELF has been shown to track fibrosis response to treatment, ELF could be repeated to monitor disease progression and function as a biofeedback marker to support abstinence. Patients with ‘moderate risk’ scores (8.3-10.49) should be counselled regarding the possibility of significant liver damage due to alcohol. In patients with an ELF 9.8-10.49, orthogonal tests may be considered. Patients with ‘high risk’ ELF≧10.5 should be referred to secondary care for further investigations and consideration given to monitoring for sequelae of cirrhosis.

This model is a conservative introduction of ELF into the primary care setting and should be implemented in conjunction with clinical judgement. It is important not to over-estimate its utility; no diagnostic test is without fault and we do not advocate its use to exclude those with a high clinical suspicion of significant ARLD from escalation of care. We also do not recommend use of ELF to screen without clinical indication, given normal ELF ranges from 7.0-9.9 among Caucasian males. This pathway may eventually validate a sufficiently sensitive ELF score to exclude cirrhosis or advanced fibrosis in primary care.
