# Supplementary material for: The Enhanced Liver Fibrosis test maintains its diagnostic and prognostic performance in alcohol-related liver disease: a cohort study
Source: BMC Gastroenterol. 2021 Jun 28;21:268. doi: 10.1186/s12876-021-01795-5 (PMC8240375; doi:10.1186/s12876-021-01795-5)
Supplement: Supplementary file 3 — Additional file 3. Unadjusted Hazard Ratios, and Hazard Ratios adjusted for age and sex, for liver related events are presented for Cohorts 2 and 3 for ELF as a continuous variable, for ELF at different cut-offs and for Ishak categories. AUROC for liver related events and all-cause mortality are presented for ELF and histological staging for Cohort 2 and Cohort 3. The numbers of subjects in the cohorts were insufficient to allow meaningful Kaplan-Meier Survival analyses or logistic regression analyses for cohorts 2 or 3. [file 12876_2021_1795_MOESM3_ESM.docx]

|  | **ELF cut- offs** | **Unadjusted HRs (95% CI)** | **P value** | **HRs adjusted for age and sex (95% CI)** | **P value** |
| --- | --- | --- | --- | --- | --- |
| **ELF as continuous (overall)** | n/a | 1.55 (1.27-1.89) | <0.001 | 1.54 (1.25-1.90) | <0.001 |
| **ELF split into four categories** | <9.8 (reference)  9.8-10.49  10.5-11.29  ≧11.3 | 1  2.23 (0.37-13.38)  4.93 (0.90-26.93)  9.89 (2.21-44.20) | 0.380  0.66  <0.01 | n/a  1.49 (0.23-9.82)  3.36 (0.57-19.60)  8.441 (1.82-39.14) | 0.676  0.179  <0.01 |
| **ELF split into two categories** | <10.5  ≧10.5 | 1  5.31 (1.95-14.47) | <0.005 | 1  4.98 (1.81-13.66) | <0.01 |
| **Ishak split into three categories** | <3 (reference)  3-4  5-6 | 1  1.92 (0.27-13.64)  7.11 (1.64-30.77) | 0.514  <0.01 | 1  1.85 (0.259-13.16)  6.71 (1.54-29.25) | 0.540  <0.05 |
| **Ishak split into two categories** | 0-4  5-6 | 1  5.41 (1.82-16.05) | <0.01 | 1  5.16 (1.73-15.42) | <0.01 |

**Hazard Ratios - Cohort-2 only**

**Hazard Ratios - Cohort-3 only**

|  | **ELF cut- offs** | **Unadjusted HRs (95% CI)** | **P value** | **HRs adjusted for age and sex (95% CI)** | **P value** |
| --- | --- | --- | --- | --- | --- |
| **ELF as continuous (overall)** | n/a | 1.12 | 0.253 | 1.47 (1.37-2.09) | <0.05 |
| **ELF split into four categories** | <9.8 (reference)  9.8-10.49  10.5-11.29  ≧11.3 | 1  n/a  21.05 (1.02-433.0)  5.47 (0.66-44.90) | <0.05  0.114 | n/a  n/a  24.46 (1.06-566.0)  25.42 (1.50-418.9) | <0.05  <0.05 |
| **ELF split into two categories** | <10.5  ≧10.5 | 1  5.67 (0.70-46.18) | 0.105 | 1  25.07 (1.77-354.4) | <0.05 |
| **Ishak split into three categories** | <3 (reference)  3-4  5-6 | 1  123.33 (0.64-237516.98)  28.44 (0.20-39475.4) | 0.212  0.365 | 1  n/a  n/a |  |
| **Ishak split into two categories** | 0-4  5-6 | 1  2.26 (0.29-17.77) | 0.437 | 1  8.29 (0.68-104.65) | 0.102 |

**AUROC for liver related events and all cause mortality -** Cohort 2

|  | AUROC at 6 years (95% CI) | AUROC at 7 years (95% CI) | AUROC at 8 years (95% CI) |
| --- | --- | --- | --- |
| **Liver Related Event** | | | |
| ELF | 0.765 (0.629-0.901) | 0.801 (0.678-0.924) | 0.806 (0.686-0.927) |
| Biopsy | 0.681 (0.532-0.830) | 0.721 (0.578-0.864) | 0.742 (0.602-0.881) |
| p-value* | 0.209 | 0.208 | 0.2939 |
| **All-Cause Mortality** | | | |
| ELF | 0.719 (0.567-0.871) | 0.756 (0.616-0.897) | 0.756 (0.616-0.897) |
| Biopsy | 0.621 (0.458-0.784) | 0.663 (0.507-0.820) | 0.663 (0.507-0.820) |
| p-value* | 0.171 | 0.163 | 0.163 |

**AUROC for liver related events and all cause mortality -** Cohort 3

|  | AUROC at 6 years (95% CI) | AUROC at 7 years (95% CI) | AUROC at 8 years (95% CI) |
| --- | --- | --- | --- |
| **Liver Related Event** | | | |
| ELF | 1.00 (1.00-1.00) | 1.00 (1.00-1.00) | 1.00 (1.00-1.00) |
| Biopsy | 0.692 (0.195-1.00) | 0.692 (0.195-1.00) | 0.692 (0.195-1.00) |
| p-value* | 0.320 | 0.320 | 0.320 |
| **All-Cause Mortality** | | | |
| ELF | 0.722 (0.455-0.990) | 0.589 (0.280-0.898) | 0.589 (0.280-0.898) |
| Biopsy | 0.481 (0.176-0.787) | 0.357 (0.065-0.649) | 0.357 (0.065-0.649) |
| p-value* | 0.115 | 0.120 | 0.120 |

*****P-values determined by De Long’s method.

Insufficient patient numbers if splitting cohorts to complete meaningful Kaplan-Meier Survival or Logistic regression analyses for cohort 2 or cohort 3.
